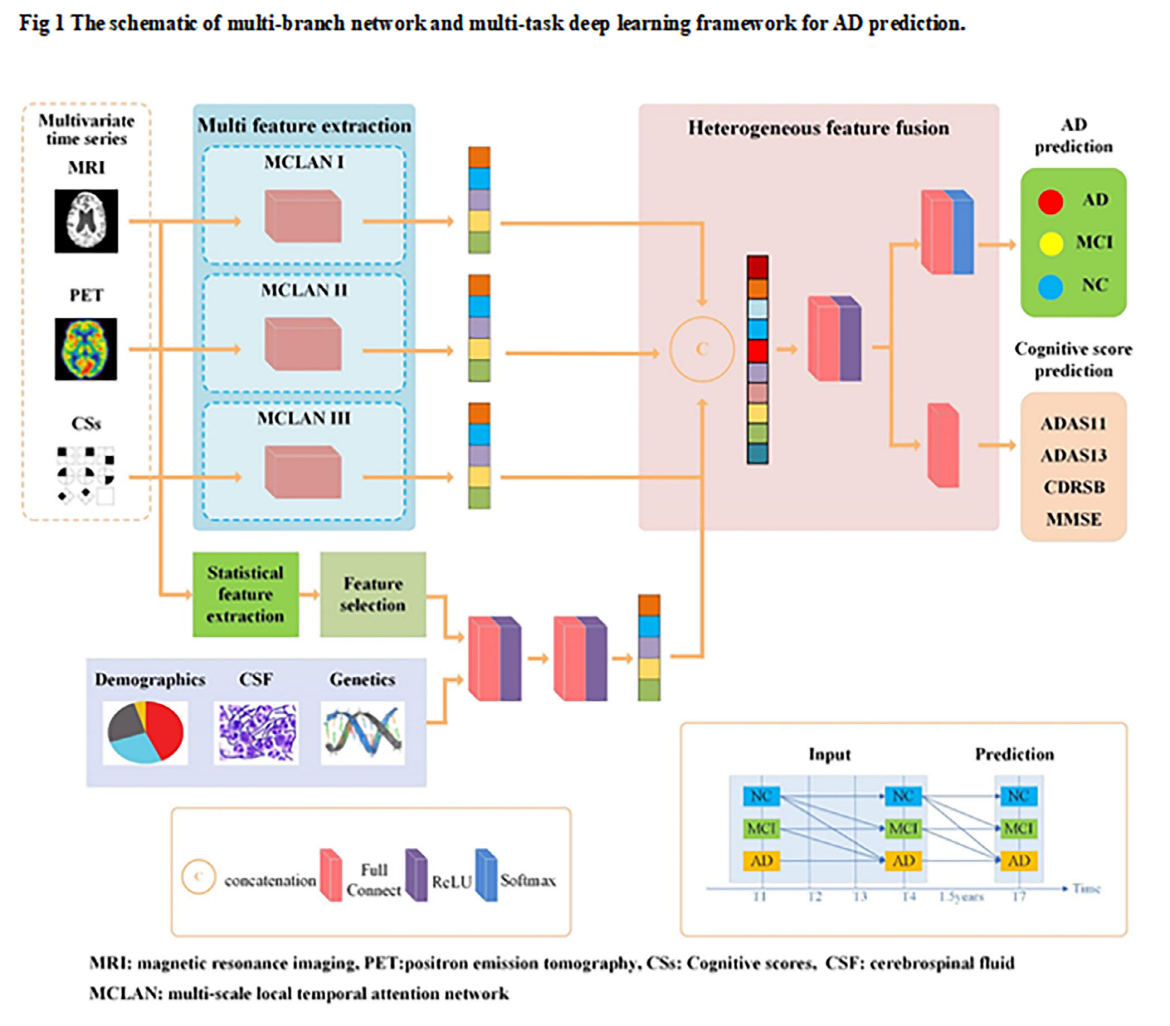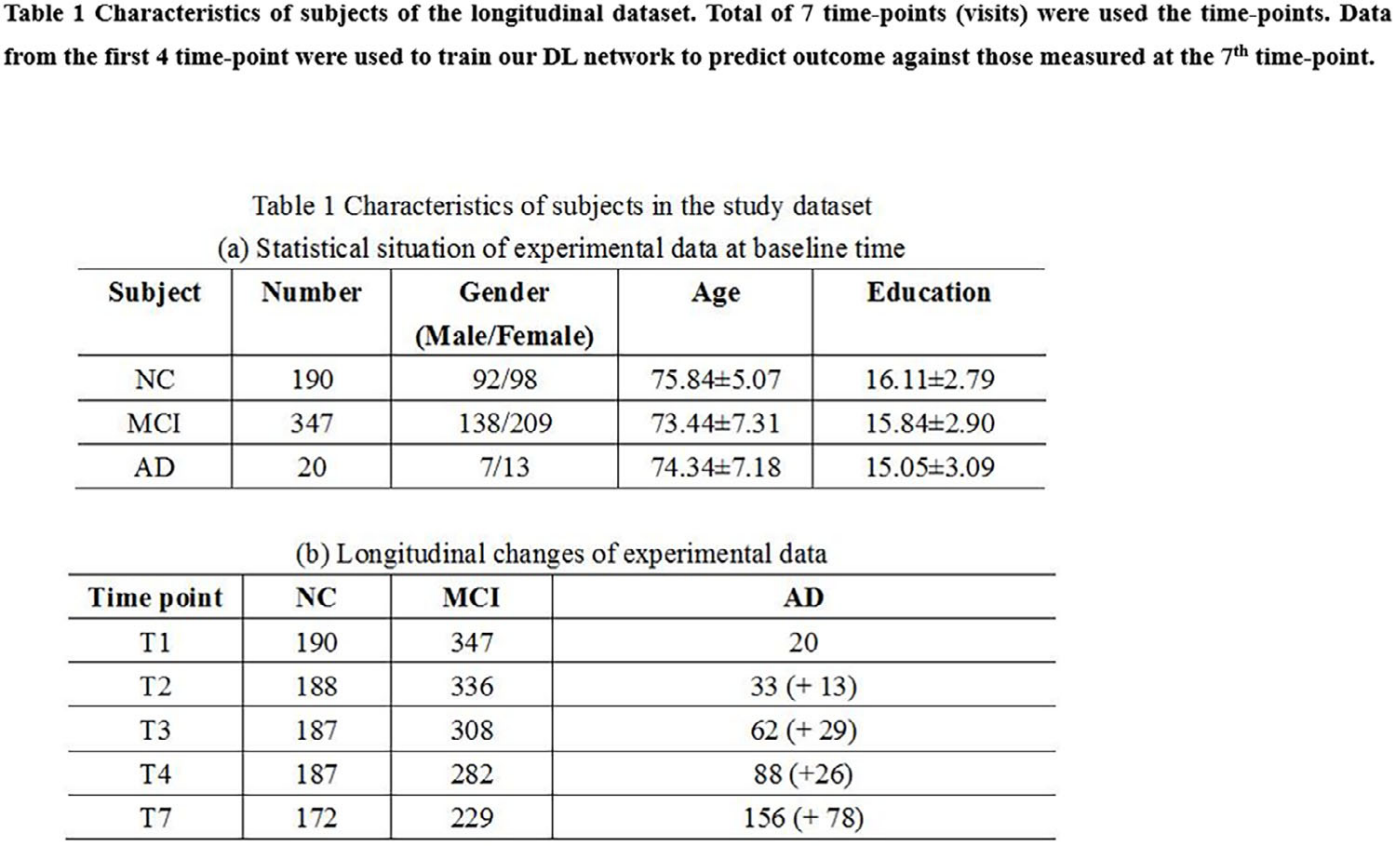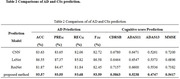# Development of AI‐4‐AD tools for evaluation and prognosis of AD conversion: multi‐branch network and multi‐task deep learning algorism

**DOI:** 10.1002/alz.092964

**Published:** 2025-01-03

**Authors:** Liang Han, Ran Pang, Qing Ren, Xiujuan Pu, Yuan Liu, Yuanfan Tan, Qing X Yang

**Affiliations:** ^1^ School of Microelectronics and Communication Engineering, Chongqing University, Chongqing China; ^2^ Penn State University College of Medicine, Hershey, PA USA

## Abstract

**Background:**

AD prevention and early interventions require tools for evaluation of people during aging for diagnosis and prognosis of AD conversion. Since AD is a complicated continuum of neurodegenerative processes, developing of such tools have been difficult because it needs longitudinal and multimodal data which are often complicated and incomplete. To address this challenge, we are developing AI4AD framework using ADNI data.

**Method:**

As shown in Fig. 1, we used the multimodal features of subjects in the initial four time points to predict the disease status after 1.5 years. The informative feature set with available multi‐modal data (MRI, PET and cognitive score (CSs)), is extracted as a multivariate time series. Then, a multi‐scale local temporal attention network (**MCLAN**) is constructed to extract corresponding **deep features** from three categories of multivariate time series respectively. Furthermore, a set of statistical features are extracted from multivariate the time series. Subsequently, fusion of selected deep features, statistical features and background knowledge (demographics, CSF, genetics feature) at baseline are performed using a **DL feature network**. Finally, AD and CSs predictions are realized utilizing multi‐task DL algorism with Adam optimization algorithm and the five‐fold cross‐validation method. 190 CN subjects, 347 MCI subjects and 20 AD subjects were selected according to the first visit diagnosis from TADPOLE database (Table 1a). The longitudinal data for training and ground truth of AD conversion are shown in Table 1(b).

**Result:**

The accuracy (ACC), accuracy (PRE), recall (REC) and F_1_ score of the proposed AD prediction method in the AD classification were 93.57%, 93.55%, 93.68%, and 93.59%, respectively, which is greatly improved the predictive ability of those by the conventional methods (Table 2). In addition, the root mean square error (RMSE) on the CSs prediction of CDRSB, ADAS11, ADAS13 and MMSE are 0.5863, 0.5238, 0.4747 and 0.5417 respectively.

**Conclusion:**

Our DL method consolidates multi‐modal medical information at different visits to predict the disease’s progression, which greatly improved AD prognosis from those using traditional DL algorithms (CNN, LeNet and ResNet). The AI4AD framework proposed here can also be tailored to other diseases with similar data characteristics.